# Initial DNA Interactions of the Binuclear Threading Intercalator Λ,Λ-[μbidppz(bipy)_4_Ru_2_]^4+^: An NMR Study with [d(CGCGAATTCGCG)]_2_**

**DOI:** 10.1002/chem.201203175

**Published:** 2013-02-28

**Authors:** Lisha Wu, Anna Reymer, Cecilia Persson, Krzysztof Kazimierczuk, Tom Brown, Per Lincoln, Bengt Nordén, Martin Billeter

**Affiliations:** [a] Department of Chemical and Biological Engineering, Chalmers University of Technology41296 Gothenburg (Sweden), Fax: (+46) 31-772-3858 E-mail: norden@chalmers.se; [b] Swedish NMR Center, University of Gothenburg40520 Gothenburg (Sweden); [c] Faculty of Chemistry, University of Warsaw02093 Warsaw (Poland); [d] School of Chemistry, University of SouthamptonSO17 1BJ (UK); [e] Department of Chemistry and Molecular Biology, University of Gothenburg40530 Gothenburg (Sweden)

**Keywords:** DNA, intercalation, minor-groove binding, NMR spectroscopy, ruthenium

## Abstract

Binuclear polypyridine ruthenium compounds have been shown to slowly intercalate into DNA, following a fast initial binding on the DNA surface. For these compounds, intercalation requires threading of a bulky substituent, containing one Ru^II^, through the DNA base-pair stack, and the accompanying DNA duplex distortions are much more severe than with intercalation of mononuclear compounds. Structural understanding of the process of intercalation may greatly gain from a characterisation of the initial interactions between binuclear Ru^II^ compounds and DNA. We report a structural NMR study on the binuclear Ru^II^ intercalator Λ,Λ-B (Λ,Λ-[μ-bidppz(bipy)_4_Ru_2_]^4+^; bidppz=11,11′-bis(dipyrido[3,2-*a*:2′,3′-*c*]phenazinyl, bipy = 2,2′-bipyridine) mixed with the palindromic DNA [d(CGCGAATTCGCG)]_2_. Threading of Λ,Λ-B depends on the presence and length of AT stretches in the DNA. Therefore, the latter was selected to promote initial binding, but due to the short stretch of AT base pairs, final intercalation is prevented. Structural calculations provide a model for the interaction: Λ,Λ-B is trapped in a well-defined surface-bound state consisting of an eccentric minor-groove binding. Most of the interaction enthalpy originates from electrostatic and van der Waals contacts, whereas intermolecular hydrogen bonds may help to define a unique position of Λ,Λ-B. Molecular dynamics simulations show that this minor-groove binding mode is stable on a nanosecond scale. To the best of our knowledge, this is the first structural study by NMR spectroscopy on a binuclear Ru compound bound to DNA. In the calculated structure, one of the positively charged Ru^2+^ moieties is near the central AATT region; this is favourable in view of potential intercalation as observed by optical methods for DNA with longer AT stretches. Circular dichroism (CD) spectroscopy suggests that a similar binding geometry is formed in mixtures of Λ,Λ-B with natural calf thymus DNA. The present minor-groove binding mode is proposed to represent the initial surface interactions of binuclear Ru^II^ compounds prior to intercalation into AT-rich DNA.

## Introduction

**Scheme 1:**
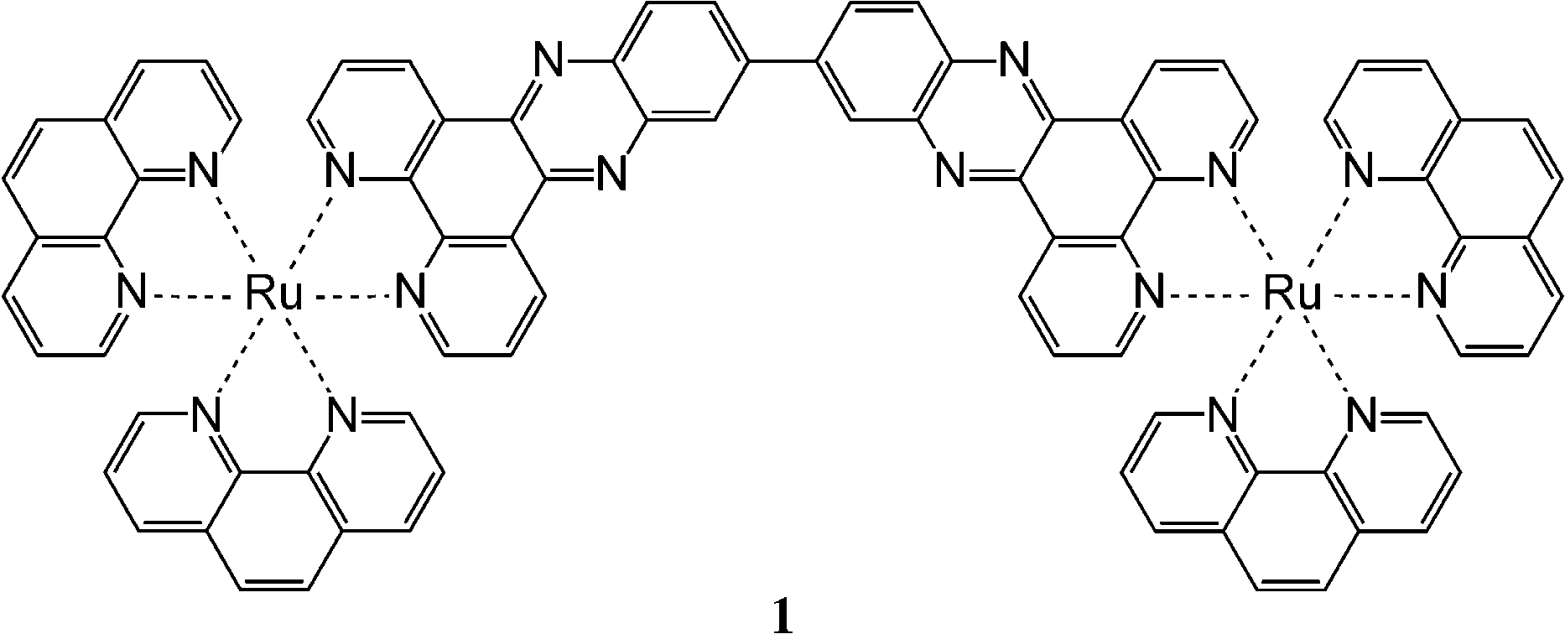
Chemical structure of [μ-bidppz(phen)_4_Ru_2_]^4+^ (bidppz=11,11′-bis(dipyrido[3,2-*a*:2′,3′-*c*]phenazinyl), a semi-rigid binuclear derivative of the “light switch ” monomer [Ru(phen)_2_dppz]^2+^.

Structural investigations of interactions between DNA and small molecules are of paramount importance for developing and improving chemotherapeutic agents that recognise a specific binding site.^1^–^3^ In this context, the unique properties of octahedral-coordinated chiral (left-handed (Λ) or right-handed (Δ)) ruthenium(II) polypyridyl compounds have for many years captured great interest regarding DNA binding as optical probes, photo-reagents and inhibitors of DNA-related cellular processes.^4^–^6^ An example is the well-known monomeric prototype “light-switch” compound [Ru(phen)_2_dppz]^2+^ (the monomer of compound **1** shown in Scheme 1; phen=1,10-phenanthroline; dppz=dipyrido[3,2-*a*:2,3-*c*]phenazine), which intercalates into DNA by inserting its dppz moiety between the base pairs of DNA resulting in a thousand-fold enhancement of the luminescence brightness.^7^ Recent crystal and NMR structure studies of monomeric Ru^II^ compounds interacting with oligonucleotide duplexes suggest that intercalation is sequence dependent, and that additional binding sites on the DNA may be exploited.^8^, ^9^

**Figure 1:**
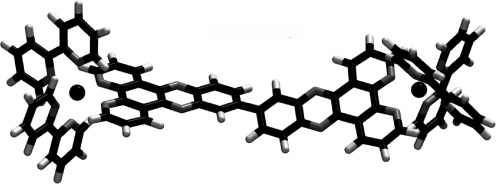
Structure of Λ,Λ-[μ-bidppz(bipy)_4_Ru_2_]^4+^ (Λ,Λ-B). Colour coding: carbon atoms black, nitrogen atoms grey and hydrogen atoms white; Ru^II^ ions are shown as spheres.

Larger as well as more positively charged binuclear compounds have been observed to be more efficient in sensing larger scale sequence-specific textural properties of the nucleic acid structure.^10^ The binuclear Ru^II^ compound [μ-bidppz(phen)_4_Ru_2_]^4+^ (**1**) (Scheme 1) and its derivatives have been shown to intercalate by inserting the bridging bidppz ligand between base pairs, leaving one Ru(phen)_2_ moiety in each groove. However, the dumbbell shape of these dimers (Figure 1) requires a very different mode of intercalation compared to the monomer: instead of sliding a planar structure, such as dppz of a monomeric Ru^II^ compound, between two base pairs, the bulky substituent surrounding a Ru^II^ ion has to be threaded through the base-pair stack; this requires severe DNA distortions and probably also transient opening of one base pair.^11^ Just like the monomer, intercalation of the bidppz ring system of binuclear Ru^II^ compounds is accompanied by a very large luminescence increase.^12^ Upon interaction with calf thymus DNA (ct-DNA) the Δ,Δ-enantiomer of **1** has been shown by linear dichroism (LD) to reorganise from an initial surface-bound geometry into an intercalative binding mode in a very slow process (two weeks at room temperature).^12^

Ru^II^ compounds such as **1** have been shown to selectively thread-intercalate into long AT-rich regions,^13^ the 2,2′-bipyridine analogue of compound **1**, Λ,Λ-[μ-bidppz(bipy)_4_Ru_2_]^4+^ (Λ,Λ-B; Figure 1) being the most discriminative.^10^ Although threading into [poly(dAdT)]_2_ occurs within minutes, this interaction rate is about 2500-fold slower with natural DNA such as calf thymus DNA. A certain length of the stretch of AT base pairs is required for fast intercalation: a dramatic drop in the threading rate was observed when the number of consecutive base pairs (bp) with alternating AT sequence was decreased from 14 to 10 bp, and no threading seems to occur for a stretch of six AT base pairs.^13^ Another significant observation concerns the intercalation rate. This is also in the best cases slow, varying at room temperature from a *t*_1/2_ of 3.4 min for [poly(dAdT)]_2_ and weeks for calf thymus DNA.^10^ Thus, the intercalation process must occur in two steps. Diffusion guided by the electrostatic attraction by the DNA phosphate groups results in the rapid formation of a complex between Λ,Λ-B and the DNA, where Λ,Λ-B is bound to the surface of the DNA, perhaps near an AT-rich region due to the local increase of the electronegative potential surrounding these regions.^14^ Provided a suitable thermodynamic interaction force is present, this is then followed by a slow re-orientation eventually yielding an intercalating state. Although this latter state has been detected by optical methods,^12^ little is known about the process of intercalation and in particular about the intermediate surface bound state. LD studies estimate that the angle between the long axis of Λ,Λ-B and the DNA helix axis is about 65°.^15^ However, no 3D structure of a binuclear Ru^II^ compound bound to DNA has been reported so far.

By characterising the intermediate, surface-bound geometry, the following questions can be addressed: in which groove does Λ,Λ-B initially bind and therefore, from which groove does it thread-intercalate into DNA; how do the bulky “propellers” of the rather rigid Λ,Λ-B (Figure 1) orient themselves with respect to the DNA, and what interactions stabilise this initial complex. Here, an NMR structural study based on NOE observations is combined with MD (molecular dynamics) simulations to test the (short-term) stability of the resulting structures. The selection of a DNA duplex was based on the following requirements: it should contain an AT-rich region that attracts Λ,Λ-B, but the sequence should not allow any intercalation, but rather trap a surface-bound intermediate state. The well-studied palindromic 12-mer [d(CGCGAATTCGCG)]_2_, originally constructed by Dickerson et al.,^16^ fulfils these demands, and offers several CG base pairs for stabilisation of the ends. Note however, that the short AT region does not allow both Ru^2+^ centres of Λ,Λ-B to simultaneously approach this central stretch. Thus, the additional question arises as to how the two Ru^2+^ centres adjust to the presence of only four A–T base pairs.

## Results

**Figure 2:**
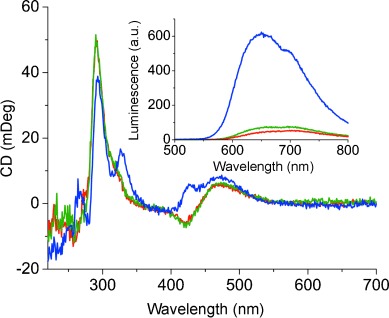
Circular dichroism spectra of mixtures with 50 μm Λ,Λ-B in 20 mm phosphate buffer (pH 6.5), 100 mm NaCl water solution: [d(CGCGAATTCGCG)]_2_ (green curve), ct-DNA (red curve, mostly covered by the green curve) and [poly(dAdT)]_2_ (blue curve). The [nucleotides]/[Λ,Λ-B] ratio is 24:1 for all mixtures. Inset: Luminescence spectra of the corresponding mixtures with the same colour coding, excitation wavelength: *λ*=410 nm. All spectra were recorded about half an hour after mixing at 25°.

**Optical spectroscopy**: Circular dichroism (CD) and luminescence spectra were compared for various DNA sequences mixed with Λ,Λ-B (Figure 2). At room temperature, Λ,Λ-B thread-intercalates into [poly(dAdT)]_2_ within less than 5 min, but only very slowly into calf thymus DNA (ct-DNA).^12^, ^15^, ^17^ The CD spectra of both [d(CGCGAATTCGCG)]_2_ (Figure 2, green curve) and ct-DNA immediately after mixing (Figure 2, red curve) with Λ,Λ-B are strikingly similar, but differ strongly from the CD spectrum of the [poly(dAdT)]_2_/Λ,Λ-B mixture (Figure 2, blue curve).^15^ These pronounced spectral differences suggest that Λ,Λ-B binds to [d(CGCGAATTCGCG)]_2_ in a similar way as the initial binding to ct-DNA.^18^ Examination of the same samples with fluorescence spectroscopy shows a six times lower magnitude of luminescence intensity for both ct-DNA and [d(CGCGAATTCGCG)]_2_ when compared to [poly(dAdT)]_2_, the reduced luminescence reflecting access to solvent water for externally bound complex,^12^ thus supporting the conclusion from the CD data.

**Conditions for NMR measurements**: Comparison of NOESY spectra for samples with a 1:1 mixture of [d(CGCGAATTCGCG)]_2_ and Λ,Λ-B, in 100 mm NaCl solution or in 100 mm NaCl solution with 20 mm sodium phosphate buffer (pH 6.5), showed that without buffer several imino proton peaks for the GC base pairs were missing and several cross peaks became weaker than the corresponding ones in buffered solution (Figure S1 in the Supporting Information). Therefore, 20 mm sodium phosphate buffer with 100 mm NaCl, pH 6.5, was utilised in all samples. When titrating Λ,Λ-B into a DNA solution starting from a ratio of 0:1 to a final ratio of 1:1, new resonances were appearing consistent with Λ,Λ-B binding to DNA. With further titration, the excess of Λ,Λ-B yielded strong line broadening, preventing observations of any other changes in the spectrum (Figure S2 in the Supporting Information). Therefore, a 1:1 ratio of Λ,Λ-B and [d(CGCGAATTCGCG)]_2_ was selected for all 2D NMR experiments. It proved necessary to add Λ,Λ-B to the DNA solution; the reverse order resulted already at very low concentrations of DNA in precipitation and disappearance of signals.

**Assignment of free DNA and free Λ,Λ-B**: ^1^H NMR resonances of free [d(CGCGAATTCGCG)]_2_ were assigned by using standard techniques.^19^, ^20^ Five imino proton resonances were observed, indicating that the oligonucleotide forms a stable double helix at the given conditions, with fraying indications only for the first and last base pairs, observations that are in agreement with previous studies.^19^, ^21^ A B-type conformation is confirmed by the position of the two most downfield-shifted resonances, the imino protons for the thymine bases (*δ*=13.75 and 13.61 ppm), followed by those of the guanine bases (*δ*=13.05, 12.88 and 12.68 ppm).^22^ Comparison of the chemical shifts with literature values^23^ reveals only small deviations (Table S1 in the Supporting Information).

**Figure 3:**
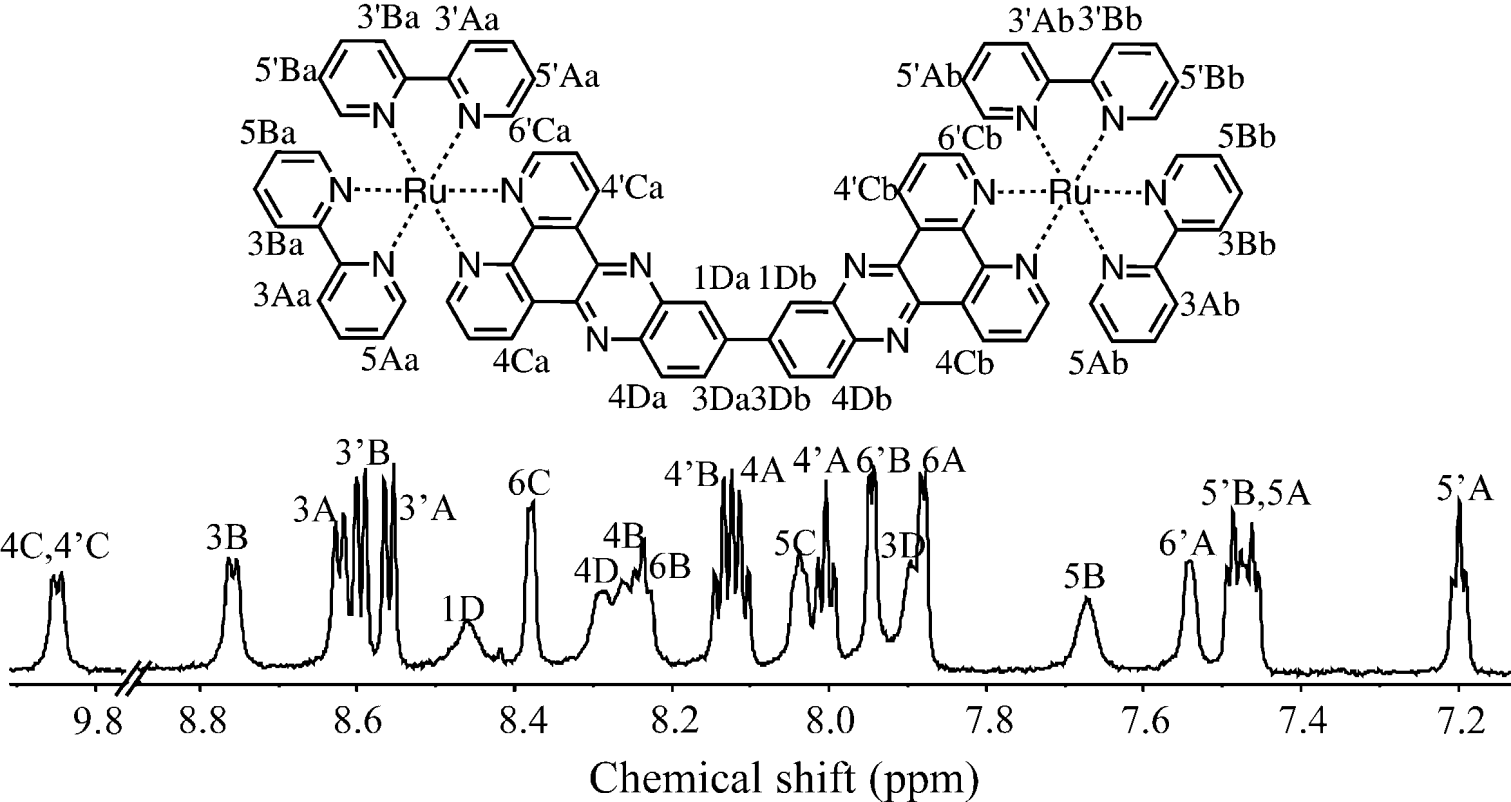
^1^H NMR assignments of 1 mm Λ,Λ-B in 20 mm phosphate, pH 6.5, 100 mm NaCl water solution. Resonance assignments refer to positional symbols defined in the chemical structure of Λ,Λ-B with positional symbols at the carbon atoms at the top. Initial numbers (with or without primes) indicate the position within the rings, which are identified by capital letters. “A” and “B” are the axial and equatorial (with respect to the dppz plane) pyridine rings of the bipyridine ligands, respectively; “C” denotes the pyridine and “D” denotes the benzene rings of the dppz ligand. Primed and non-primed numbers discriminate the two bipyridine systems attached to the ruthenium atoms. Lower case letters define the two equivalent monomer units.

Free Λ,Λ-B in 20 mm phosphate buffer solution at room temperature was assigned by analysing TOCSY and NOESY spectra. One three-spin system and four four-spin systems were detected. Figure 3 (lower panel) shows the resonance assignment in a 1D spectrum (chemical shifts are listed in Table S2 in the Supporting Information). Due to the overall two-fold symmetry, both monomer units, labelled “a” and “b” in Figure 3, are identical. The two axial (A, A′) and the two equatorial (B, B′) pyridine rings show slightly different chemical shifts due to the breaking of the monomer two-fold symmetry by the pivot bond, however, the two pyridine rings (C and C′) of the bidppz ligand appear identical. The extraordinary downfield-shifted peak (*δ*=9.86 ppm) was assigned to H4C/H4′C because of their close distance to the electron-withdrawing nitrogen atoms of the phenazine part of the bidppz ligand.

**Figure 4:**
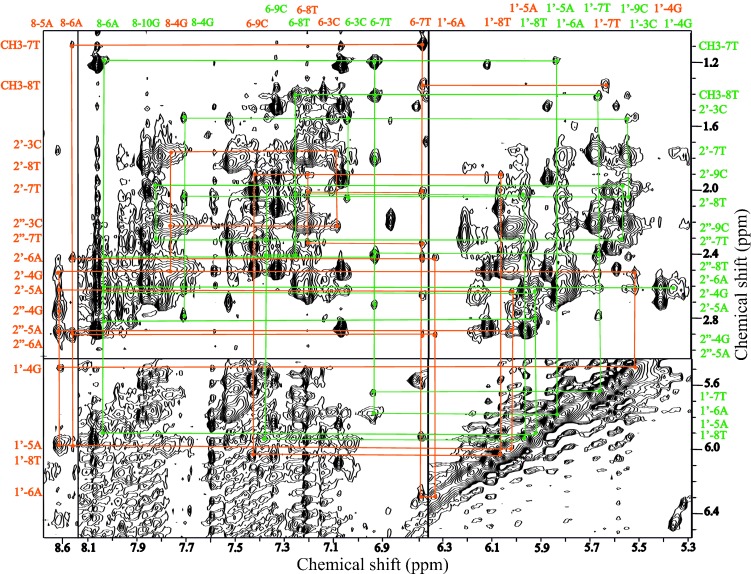
Selected regions of the NOESY spectrum of a 1:1 mixture of 1 mm Λ,Λ-B with [d(CGCGAATTCGCG)]_2_, showing connections between base and sugar protons. Sequential walks are presented for the α strand (green) and the β strand (orange). Individual cross peaks are identified on the left and top borders with numbers indicating the positions in the sugar or base rings, followed by the nucleotide number and type.

**Figure 5:**
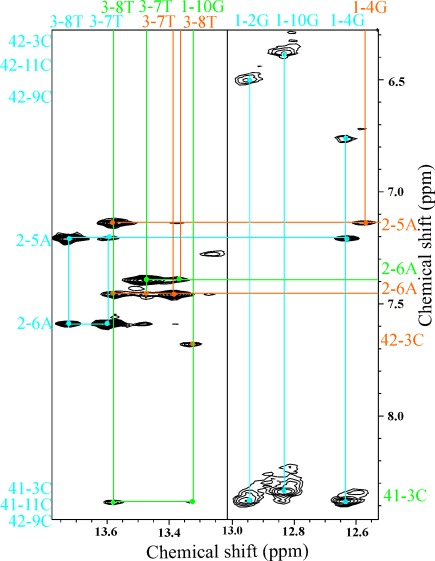
Imino region of the NOESY spectrum shown in Figure 4. Cross-peak assignments for all three observed DNA strands are given with numbers indicating the position in the base rings, followed by the nucleotide number and type: symmetric DNA (blue), asymmetric DNA with green (α strand) and orange (β strand). Vertical and horizontal lines connect peaks to the assignment texts.

**Assignments of bound DNA and bound Λ,Λ-B**: Upon interaction between Λ,Λ-B and [d(CGCGAATTCGCG)]_2_, the DNA signals are split into three sets, each of which can be assigned to an individual, complete or nearly complete DNA strand. The strongest signals can be assigned to a symmetric duplex, very similar to the one for free DNA, and a basically complete assignment was achieved. Besides this symmetric DNA duplex, somewhat weaker signals characterise two additional strands with almost complete assignment (in the following referred to as α strand and β strand), which together form an asymmetric DNA duplex based on interstrand NOEs. Sequential walks for these two strands are shown in Figure 4: green for the α strand and orange for the β strand (sequential and interstrand NOEs are identified in the structure of Figure S3 in the Supporting Information). On average, the β strand shows stronger chemical shift differences from free DNA than the α strand (Figure S4 in the Supporting Information). In Figure 5, the region with the imino proton signals is shown for all three DNA strands, by using the same colour coding as in Figure 4 for the asymmetric strands. Although these NOE networks are incomplete, lacking connections to the terminal nucleotides, their observation clearly excludes the possibility of intercalation anywhere between base pairs 2 and 9 (a complete list of the chemical shifts for all DNA strands as well as further assignment information is given in Table S1 in the Supporting Information, and chemical shift differences of the α strand and the β strand are shown in Figure S4 in the Supporting Information).

In the NMR spectra of the complex between Λ,Λ-B and [d(CGCGAATTCGCG)]_2_, four resonances of Λ,Λ-B are found with chemical shifts *δ*>9 ppm, that is, outside the spectral regions occupied by DNA resonances (Figure S7 in the Supporting Information). Two resonances exhibit intermolecular NOEs (see below) to the DNA: one appears at *δ*=9.35 and the other at 9.14 ppm. Based on the patterns of intramolecular NOESY and TOCSY peaks and chemical shift differences to the resonances of free Λ,Λ-B (for details see Section 3 in the Supporting Information), these four resonances can only be assigned to the H4Ca, H4′Ca, H4Cb and H4′Cb protons; these are expected to have individual resonances after symmetry breaking. Unambiguous individual assignments of the four atoms to these resonances are not possible at this stage, and have to await structural calculations.

**Figure 6:**
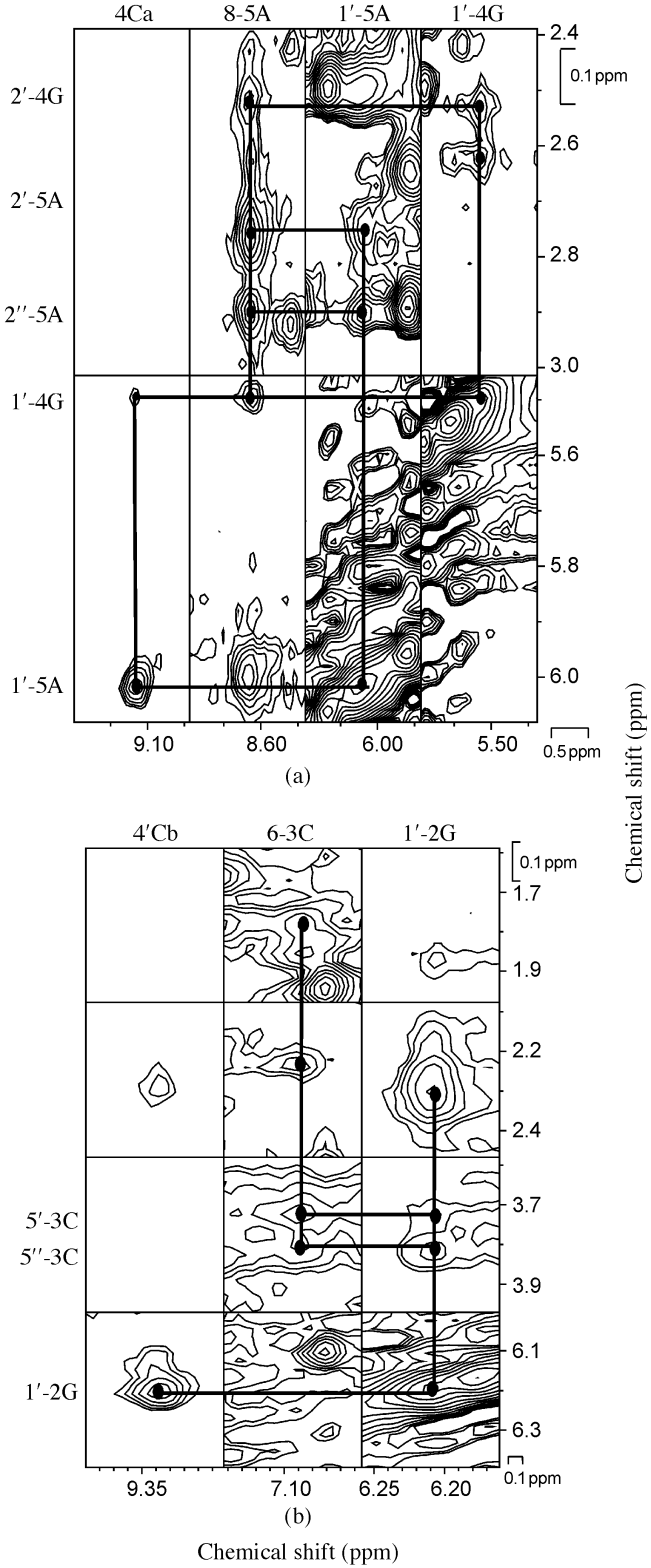
Spectral regions from the NOESY spectra shown in Figures 4 and 5. a) NOEs between the Λ,Λ-B proton 4Ca (see Figure 3) at *δ*=9.14 ppm to H1′ of 4G and H1′ of 5A. b) NOE between the Λ,Λ-B proton 4′Cb at *δ*=9.35 ppm to H1′ of 2G. All NOEs are to the β strand. To confirm the DNA assignments, these intermolecular NOEs are connected to the intramolecular network of the NOEs of the DNA. The cross peaks are identified on the left and top borders.

**Intermolecular NOESY cross peaks and CYANA calculations**: The assignment of the DNA resonances for intermolecular NOEs between Λ,Λ-B and the asymmetric DNA duplex is illustrated in Figure 6: Each NOE is connected to the network of intramolecular DNA NOEs used for the DNA assignments (Figure 4). As discussed above, only the H4Ca/b and H4′Ca/b protons remain as candidates for the assignment of the two resonances at *δ*=9.35 and 9.14 ppm in the spectra of the mixture between Λ,Λ-B and [d(CGCGAATTCGCG)]_2_. From all possible assignment combinations (four available protons for each resonance yield in total sixteen possibilities), the four with the same proton assigned to both resonances can immediately be excluded. The remaining twelve combinations consist of identical pairs due to the symmetry of Λ,Λ-B. For the remaining six combinations, CYANA calculations were performed. For one assignment combination, the CYANA calculations show no violations of the distance restraints or of any van der Waals limits exceeding 0.1 Å combined with CYANA target functions as low as 0.13. The other five assignment combinations resulted in violations of both distance and van der Waals restraints of at least 0.35 Å, and target functions exceeding 0.43 (Table S3 in the Supporting Information). Thus, the assignment with smallest violations and lowest target function was accepted, with the following three NOEs: proton 4Ca of Λ,Λ-B to the H1′ protons of both G4 and A5 (β strand), and proton 4′Cb of Λ,Λ-B to the H1′ proton of both G2 (β strand). The resulting five best structures calculated with CYANA (see the Experimental Section) by using these three NOEs show that Λ,Λ-B is aligned with the minor groove with the two monomers in an *anti* conformation with a torsion angle of 33°.

**Model structure refinement with molecular dynamics**: The resulting CYANA model was subjected to exhaustive MD simulations with the aim of further refining the structure, to evaluate its (short-term) stability and to confirm the presence of a stable minor-groove binding mode of Λ,Λ-B that satisfies, on a time average, the intermolecular NOEs. The best of the five resulting CYANA structures with the accepted NOE assignment served as starting structure for ten fully unrestrained MD runs, each 5 ns long, after equilibration and REMD (see the Experimental Section). Consistency with the NOE restraints was tested by calculating for each observed intermolecular NOE an average distance (*d*_NOE_) between the two protons according to Equation (1) (see the Experimental Section). Table 1 provides comparisons between the NOE distance limits and the corresponding average distances *d*_NOE_ for each MD run. Complete consistency is confirmed for five runs (4–7 and 10). For run 2, the maximal violation does not exceed 0.1 Å. Remarkably, for all ten runs the two shorter restraints with limits of 3.5 Å are fulfilled on a time average (with the above small exception), and only the longer restraint is sometimes violated. This latter violations result from either a small displacement of the Λ,Λ-B along the minor groove or small changes in the DNA structure. In one simulation (run 3), structural divergence indications become apparent towards the end of the run.

**Table 1:** Assigned NOEs used for the structural calculations. DNA signals are all from the β strand. Distances *d*_NOE_ according to Equation (1) are given for the ten unrestrained MD runs of 5 ns each. The five runs that are strictly consistent with the NOEs are at positions 4–7 and 10.

NOE	DNA^[a]^	Λ,Λ-B	Limit^[b]^	*d*_NOE_ in 10 MD runs [Å]									
1	G4	4CA	5.0	5.4	4.2	5.7	4.9	5.0	4.6	4.6	5.5	5.7	4.6
2	A5	4CA	3.5	2.7	2.8	2.9	2.7	2.8	2.7	2.8	3.1	2.8	2.7
3	G2	4′CB	3.5	3.2	3.6	3.4	3.0	3.1	3.0	2.9	3.5	2.4	2.9

[a] H1′ protons from the β strand of the DNA. [b] Distance restraints from NOE observations in [Å].

**3D structure of the DNA–Λ,Λ-B complex**: The fact that at least five runs are stable over 5 ns of unrestrained MD strongly supports the existence of a stable complex with Λ,Λ-B bound to the minor groove of the DNA in a manner consistent with all NOE restraints. For structural comparisons, averaged structures were determined from the snapshots of each MD. The relative positions of Λ,Λ-B in the different MD trajectories were then compared as follows: the average structures of each MD run were superimposed for minimal root-mean-square deviation (RMSD) of the most stable parts of the binding half of the DNA, that is, base pairs 3–6 (all heavy atoms). Following this superposition the average displacement of Λ,Λ-B heavy atoms was (0.6±0.3) Å. Visual inspection shows that most variations affect the outer bipyridine rings. This well-defined position of Λ,Λ-B in the minor groove is supported by the identification of two hydrogen bonds (length <2.6 Å, angle <25°) in all five structures connecting Na and N′b, of Λ,Λ-B (see Figure 1) with the amino groups of the guanine bases 4 and 2, respectively.

**Figure 7:**
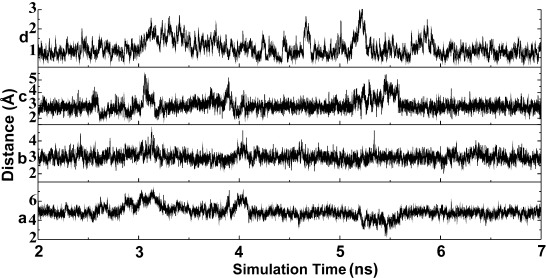
Averaged structural variations versus simulation time for MD run 7. Only the last unrestrained 5 ns are shown. The bottom three panels show the time evolution for proton–proton distances corresponding to the measured NOEs: a), b) and c) correspond to NOE 1, 2 and 3 in Table 1, respectively. d) Average displacement of heavy atoms of Λ,Λ-B between the snapshot structures and the mean structure of the corresponding trajectory interval. For these displacements, the DNA heavy atoms of base pairs 3–6 have been superimposed, followed by calculation of position differences for only the heavy atoms of Λ,Λ-B.

**Figure 8:**
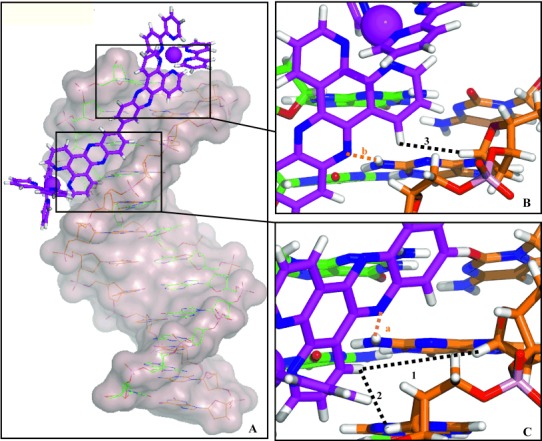
Average DNA–Λ,Λ-B structure of the 5 ns productive, unrestrained trajectory from the same MD simulation as shown in Figure 7. A) Overall side view. B,C) Details of the short distances of three intermolecular NOEs shown in Table 1. Colour coding: green=strand α, orange=strand β. Dotted lines indicate NOEs (black, numbered 1–3) and potential hydrogen bonds (orange, labelled “a” and “b”: Na and N′b of Λ,Λ-B with the amino groups of the guanines bases 4 and 2, respectively.

The average structure of run 7 yielded the smallest Λ,Λ-B displacements to the other average structures, and is therefore a good representative of all MD runs. Its Λ,Λ-B heavy atom displacements and the variations of the distances corresponding to the NOEs during the unrestrained 5 ns MD run is presented in Figure 7, demonstrating again the stability of the complex. Figure 8 shows the location of Λ,Λ-B in the minor groove of the DNA of this average structure. The angle between the main axis of the dimer and the DNA helix axis is consistent with the LD value of about 65°.^15^ One Ru^2+^ moiety is near the central AATT region. This binding geometry is secured by the hydrophobic alignment of the bridging bidppz ligands of the Λ,Λ-B with the furanose rings of the DNA backbone, as well as by electrostatic interactions of the positively charged peripheral hydrogen atom of the ligand with the negatively charged phosphate groups of the DNA backbone.

The binding of Λ,Λ-B to the DNA also results in chemical shift changes of the protons of the DNA, the largest four exceeding 0.35 ppm (Figure S4 in the Supporting Information). The H1′ of G2 is in the structure of Figure 8 located at a distance of 4.4 Å from the centre of the Λ,Λ-B ring with atoms *′Cb (Figure 3), and the angle between the connection of this proton to the ring centre and the ring plane is 7°. This position near the ring plane and close to the ring explains the shift change as a ring current effect. The H8 protons of A5 and A6 as well as the H6 proton of T7 on the other hand are in the major groove and at distances of 6.7, 7.2 and 9.0 Å of the closest Λ,Λ-B ring; the corresponding angles are between 30 and 60°. This excludes a ring current effect. However, changes of approximately 0.5 ppm for base protons in the major groove upon binding of a minor-groove ligand have been observed earlier and are probably due to conformational changes of the DNA.^24^–^26^

The interaction enthalpy was estimated by summing intermolecular van der Waals and Coulomb interactions, and by adding contributions from the two hydrogen bonds. With calculations as described in the Experimental Section, one obtains estimates of −(64.5±6.3) and −(83.2±0.33) kcal mol^−1^ for van der Waals and Coulomb interactions, respectively (averaged over the average structures of the five stable and NOE-consistent MD runs); the two hydrogen bonds add only a few kcal mol^−1^ to this interaction enthalpy (note that this enthalpy estimate does not include any free energy contribution caused by hydrophobic effects, which for this highly hydrophobic compound Λ,Λ-B is expected to further favour complex formation). Due to the asymmetric position of Λ,Λ-B with respect to the DNA centre, one of the monomers interacts with one end of the DNA. However, only (1.6±0.6) % of the total van der Waals and Coulomb interaction enthalpy −(147.7±6.3) kcal mol^−1^ involves the DNA end surface (i.e., the surface created by cutting a 12-mer fragment from a long DNA).

## Discussion

In the NMR spectra, most of the Λ,Λ-B resonances overlap with the protons of the DNA bases at *δ*=7–8 ppm. This results in a very limited number of assignable intermolecular NOEs. However, because all observed intermolecular NOEs are from the bidppz ligand to the sugar protons of the DNA, the position of the bidppz moiety in the minor groove can be well determined. Because the flexibility of Λ,Λ-B is reduced to the single bond connecting the two monomers, the position of the bipyridine rings can be also well defined.

The present structure determination by NMR spectroscopy describes a 1:1 complex where Λ,Λ-B binds to the minor groove near one end of the asymmetric DNA duplex. The NMR data show in addition the presence of a symmetric DNA duplex (free DNA) in the NMR sample, but no additional form of Λ,Λ-B could be detected (see also the Supporting Information), with the exception of a fast exchange process located at the terminal DNA base pairs of the free DNA (Section 5 in the Supporting Information). In particular, intercalation can be excluded based on the near-complete sequential NOE connections for the asymmetric DNA duplex (Figure S3 in the Supporting Information), in agreement with the non-intercalative binding mode suggested by the CD and luminescence results. Interestingly, the NMR data exclude the presence of a significant concentration of a symmetrically bound Λ,Λ-B at the other end of the asymmetric DNA duplex (e.g., near the bottom in Figure 8 A), although no steric clash would hinder such a 2:1 complex. The complexity in our NMR spectra, with signals from symmetric and asymmetric DNA forms, precludes a precision of the resulting DNA structure that would for example allow determining conformational changes preventing binding at the other end. To our knowledge, this is the first atomic resolution structure of a complex between a binuclear Ru^II^ compound and a DNA fragment. As discussed below, we propose that this (rapidly attained) surface-binding mode approximates the starting point for the (slow) intercalation process observed with AT-rich DNA through optical measurements.^12^

Besides the obvious electrostatic attraction between Λ,Λ-B with four positive charges and the strongly negatively charged DNA, the observed minor-groove structure is also stabilised by burying the highly hydrophobic surface of Λ,Λ-B on the DNA surface, allowing two intermolecular hydrogen bonds to be formed to the phenazine nitrogen atoms. However, although these may help to define a unique position of Λ,Λ-B in the minor groove, enthalpy calculations (estimated as the sum of van der Waals and electrostatic interactions and hydrogen bonds as explained in the Experimental Section) indicate the overwhelming intermolecular attraction comes from the sequence-independent van der Waals, electrostatic and hydrophobic contributions. A similar argument shows that in spite of the location of Λ,Λ-B near one end of the DNA, the surface created by cutting the 12-mer DNA contributes negligibly (about 1.6 %) to the total interaction enthalpy. The observed preference of Ru^II^ compounds for AT-rich DNA region^13^ may explain the position of one Λ,Λ-B monomer with a central Ru ion near the AT base pairs, for which an increase of the electronegative potential was described.^14^ This results in the position of the second monomer at the end of the DNA duplex. When the upper end of the B-DNA model shown in Figure 8 is extended by four base pairs, a steric clash will result between the left wall of the minor groove and the upper left bipyridine ligand. A subsequent refinement suggests that this clash can be resolved by a slight increase of the width of the minor groove and a small tilt to the right wall of the minor groove of the upper part of the complex while still fulfilling the NOE constraints. Superposition of the upper six base pairs of the model in Figure 8 with the corresponding base pairs of the longer DNA results in a RMSD of 0.8 Å and an average displacement of the Ru^II^ compound of 1.7 Å, leaving the position and orientation of the lower Ru^II^ monomer nearly unperturbed near the AATT region.

Previous linear dichroism and luminescence studies reported that binding to the DNA surface is the first step of the slow thread-intercalation of binuclear Ru^II^ compounds. Threading intercalation is observed for various binuclear Ru^II^ compounds including Λ,Λ-B, provided a sufficiently long stretch of AT base pairs is present. The stable minor-groove binding of Λ,Λ-B to [d(CGCGAATTCGCG)]_2_, which contains a central sequence of AT base pairs that does not allow intercalation to occur, is therefore likely to correspond to a trapped intermediate prior to intercalation.

Binuclear Ru^II^ compounds differ from the better studied monomeric compounds, such as Λ-[Ru(phen)_2_dppz]^2+^ and Δ-[Ru(bpy)_2_dppz]^2+^, mostly by the need to thread the bulky substituents surrounding one Ru^II^ ion (Figure 1) through the base-pair stack. In contrast to simply sliding a planar ring, for example, dppz, between two base pair as needed in the monomeric case,^8^, ^27^ a major DNA distortion, likely combined with opening of a base pair, is required. Nonetheless, it is interesting to notice that also the monomeric compounds prefer to intercalate from the minor groove as illustrated by two recent crystallographic studies.^8^, ^27^ Besides different intercalation modes (threading between two base pairs, filling the hole created by a mismatch, entering the DNA at the fraying ends), these crystal structures also reveal subtle dependencies on the DNA sequences, for example, favouring AT over TA.

Binuclear Ru^II^ compounds such as Λ,Λ-B differ from both small molecules such as netropsin and many others,^28^ as well as proteins^29^ binding in the minor groove. Practically all DNA binding molecules utilise positive charges to offset the negative charges of the DNA backbone and the negative electrostatic potential in the grooves. On the other hand, compounds such as Λ,Λ-B are highly rigid, whereas small molecules can adapt their conformation to the curvature and shape of the bottom of the minor groove. Similarly, the flexibility of the side chains of proteins allows them to contact specific groups of the DNA bases. The ability of both small molecules and proteins to form hydrogen bonds to several hydrophilic groups of the DNA bases provides a higher potential for sequence specific DNA binding. Due to the rigidity of Λ,Λ-B, the adaptability to form hydrogen bonds is strongly reduced compared to small molecules or proteins. Λ,Λ-B and proteins share the size characteristics that prevent full integration in the minor groove. Together with the predominantly hydrophobic character and the positive charges of Λ,Λ-B, this explains the unique binding mode of Λ,Λ-B with partial-groove penetration of only the central bidppz moiety, as well as the attraction to AT-rich regions, and possibly the ability to thread-intercalate into suitable DNA.

## Conclusion

In summary, in the 1:1 DNA/Λ,Λ-B complex we have shown that Λ,Λ-B binds to the minor groove of [d(CGCGAATTCGCG)]_2_ in an asymmetric manner. General long-range electrostatic interactions are the driving force for the initial attraction between the two molecules; local variations of the electrostatic potential may then direct the Ru^II^ compound near the AT base pairs, defining the asymmetric location on the DNA. Based on estimation of binding enthalpies, a similar minor-groove binding can also be expected for other DNA sequences, including the ones with sufficiently long AT stretches that allow subsequent threading intercalation. The strikingly similar CD spectra obtained for the complex of Λ,Λ-B with [d(CGCGAATTCGCG)]_2_ and for freshly prepared mixtures of Λ,Λ-B with ct-DNA (before the slow intercalation process has taken place) indicate that a similar binding mode is present with the natural calf thymus DNA/Λ,Λ-B complex. To the best of our knowledge, this is the first NMR structure on binuclear ruthenium Λ,Λ-B interaction with DNA in a surface-bound manner. Future work will focus on a DNA sequence that allows determining the structure of a surface-bound mode before initiating threading and structural analysis of the intercalated mode.

## Experimental Section

**Materials**: The oligomer [d(CGCGAATTCGCG)]_2_ was synthesised with standard phosphoramidite chemistry, purified by HPLC, lyophilised and subsequently re-dissolved in 100 mm NaCl solution, in the presence of 20 mm sodium phosphate (pH 6.5, in the following referred to as phosphate buffer), providing stock solutions for NMR and optical spectroscopy. Calf thymus DNA (ct-DNA) and [poly(dAdT)]_2_ were obtained from Sigma–Aldrich and used as received. DNA concentrations were determined by absorbance measurements on a Cary 4B spectrophotometer, by using molar extinctions *ε*_260_=193 070 cm^−1^ m^−1^ (calculated by using OligoAnalyzer software, available online at http://www.idtdna.com) for the oligomer duplex, *ε*_260_=6600 cm^−1^ m^−1^ for ct-DNA and *ε*_262_=6600 cm^−1^ m^−1^ for [poly(dAdT)]_2_. Compound Λ,Λ-B, as tetrachloride salt, was synthesised as described elsewhere.^15^, ^17^ Its concentration was determined by using *ε*_410_=65 000 cm^−1^ m^−1^.

**Spectroscopy**: CD spectra were acquired on a Jasco J-810 spectropolarimeter by using a 1 cm quartz cell. CD samples were prepared 50 μm Λ,Λ-B in 20 mm phosphate buffer (pH 6.5), 100 mm NaCl solution. The [nucleotides]/[Λ,Λ-B] ratios are 24:1 for all mixtures, and CD spectra were recorded immediately after mixing. Emission spectra of the same samples were measured shortly after the CD scans on a Varian Eclipse spectrofluorimeter with excitation wavelength *λ*=410 nm. For all NMR measurements DNA was annealed by heating to 50 °C, where the imino signals disappeared, followed by slow cooling to 25 °C (1 °C min^−1^), where the imino signals reappeared. All samples were buffered with 20 mm sodium phosphate and the pH value was adjusted to 6.5. Spectra were recorded in Shigemi tubes with 1 mm DNA concentration in a 9:1 mixture of H_2_O/D_2_O. For the samples containing mixtures of DNA and Λ,Λ-B, the latter was added after the annealing. All NMR spectra were recorded at 25 °C on a Varian Inova 800 MHz spectrometer by using Watergate solvent suppression. One-dimensional spectra were recorded with 16 K data points; two-dimensional NOESY and TOCSY spectra were acquired by using 512 data points in *t*_1_ and 2048 data points in *t*_2_, with a pulse sequence repetition delay of 1.5 s. Both types of 2D spectra were recorded on pure DNA and on 1:1 mixture of the DNA with Λ,Λ-B, by using mixing times of 100 ms for NOESY and 80 ms for TOCSY experiments. All data were processed with NMRPipe^30^ by zero-filling to 1024 points in *t*_1_ and apodising with either a Gaussian or a shifted sine-bell function. XEASY^31^ and CCPNmr^32^ were used for analysis, assignment and peak integration of 2D spectra. 1D spectra were analysed with Mnova.^33^

**Structure calculations**: Different NOE inputs for the complex with Λ,Λ-B and [d(CGCGAATTCGCG)]_2_ were tested with CYANA^34^ calculations by using rigid B-DNA strands, whereas for the Λ,Λ-B dimer a single torsion angle was defined for the bond connecting the two monomer units (Figure 2). The two DNA strands were connected by a flexible linker comprising 14 standard CYANA linker units (these linker units consist of “pseudo-atoms” that have zero van der Waals radius); similarly, the second DNA strand was connected to the Λ,Λ-B with a 19 units long linker. A standard B-DNA double helix structure was enforced by hydrogen-bond restraints for all twelve base pairs. In addition, observed NOEs were used to define upper distance limits between Λ,Λ-B and DNA, by using upper limits of 5 Å for weak and 3.5 Å for strong NOEs. The standard CYANA protocol^34^ was applied to calculate 50 structures, of which the best five were inspected. The best model obtained from CYANA for the finally accepted NOE input was subjected to structural verification by molecular dynamics (MD) simulations performed with the AMBER11 software package.^35^ The DNA molecule was parameterised with AMBER-ff10, the Ru^II^ ion coordination sphere was obtained with an earlier described procedure,^36^, ^37^ and the AMBER GAFF force field^38^ was used for the parameterisation of the remaining atoms of Λ,Λ-B. By using standard protocols, the complex of DNA with Λ,Λ-B was solvated by explicit solvent water (TIP3P)^18^ and neutralised by sodium ions, energy minimised, heated and equilibrated during 2 ns with a series of pull-and-relax cycles, where NOE restraints were switched on and off, by means of steered MD.^39^, ^40^ Both this equilibration stage and the following productive runs were performed by using a replica-exchange MD approach (REMD)^41^ with a temperature step of 1° covering a temperature span of 300–309 K, providing an ensemble of ten parallel MD runs. The productive MD simulations used for the analysis consisted of ten trajectories of 5 ns each. These were started from the equilibrated structures, and simulated without any restraints from NOEs or for hydrogen bonds, by using constant pressure and temperature. More details on the MD simulations are provided in the Supporting Information.

**Data analysis**: Distances corresponding to observed NOEs were averaged over MD runs as given in Equation (1):


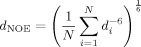
(1)

where *i* is a snapshot of the MD trajectory, *N* is the number of snapshots in this trajectory and *d_i_* is the distance in this snapshot between two protons of the NOE. The interaction energy between Λ,Λ-B and the DNA was approximated by Coulomb potentials, by using a relative permittivity of 20^42^ between the two Ru ions and the phosphates, Lennard–Jones potentials between all intermolecular pairs of atoms and hydrogen bonds between donor hydrogen atoms and acceptor atoms closer than 3 Å. For the former, point charges of +2 centred on the Ru ions and of −1 centred on the phosphorus atoms were assumed. The Lennard–Jones parameters were taken from AutoDock 3.0.5,^43^ by using the same parameters for the phosphorus (missing in the reference) as for sulfur and a cut-off of 6 Å was used. The large limit for the hydrogen-bond lengths was chosen because the structures investigated represent averaged structures over trajectories. Contributions of the intermolecular energy to the surface created by cutting the DNA (i.e., the extra surface created by extracting a 12-mer from a long DNA) consist of interactions from any Λ,Λ-B atom to the base atoms of the top base pair. For every such interaction, the angle of the interaction direction and the plane of the base were calculated: if this angle is larger than 45°, then the interaction is considered to involve the surface created by cutting the DNA (i.e., an interaction not possible in the presence of a long DNA).

## Supplementary material

As a service to our authors and readers, this journal provides supporting information supplied by the authors. Such materials are peer reviewed and may be re-organized for online delivery, but are not copy-edited or typeset. Technical support issues arising from supporting information (other than missing files) should be addressed to the authors.
