# Supplementary material for: Initial DNA Interactions of the Binuclear Threading Intercalator Λ,Λ-[μbidppz(bipy)4Ru2]4+: An NMR Study with [d(CGCGAATTCGCG)]2
Source: Chemistry. 2013 Feb 28;19(17):5401–10. doi: 10.1002/chem.201203175 (PMC3743166; doi:10.1002/chem.201203175)
Supplement: Supplementary file 1 [file chem0019-5401-SD1.pdf]

CHEMISTRY
A EUROPEAN JOURNAL

Supporting Information

© Copyright Wiley-VCH Verlag GmbH & Co. KGaA, 69451 Weinheim, 2013

Initial DNA Interactions of the Binuclear Threading Intercalator Λ,Λ-[μ-bidppz(bipy)$_4$Ru$_2$]$^{4+}$: An NMR Study with [d(CGCGAATTCGCG)]$_2$**

Lisha Wu,[a] Anna Reymer,[a] Cecilia Persson,[b] Krzysztof Kazimierczuk,[c] Tom Brown,[d] Per Lincoln,[a] Bengt Nordén,*[a] and Martin Billeter[e]

chem_201203175_sm_miscellaneous_information.pdf

Sections

1. Impact of pH, buffer on the DNA-? ,? -B mixture
2. Assignment of both symmetric and asymmetric DNA
3. Assignment of ? ,? -B and intermolecular NOEs
4. Molecular dynamic simulations
5. Other interaction modes

Section 1: Impact of pH, buffer on the DNA-? ,? -B mixture

Phosphate buffer yields some unwanted effects by showing affinity to ? ,? -B (see Section 6). Therefore, a test spectrum was recorded in the absence of any buffer. This resulted in a pH of 5, and an unstable DNA duplex with weak or missing peaks for the imino protons of the guanines. Consequently, all other spectra were recorded with 20 mM phosphate buffer at pH 6.5

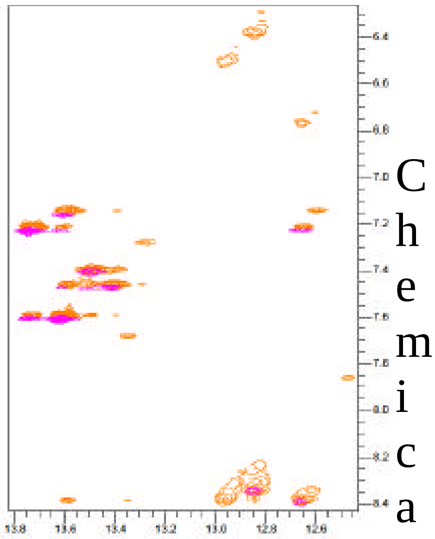

Figure S1. Two overlayed NOESY spectra with the imino proton region of 1:1 DNA-? ,? -B (1mM) mixtures. The orange spectrum was recorded at pH 6.5 with the presence of 20 mM pH=6.5 sodium phosphate buffer; the magenta spectrum was recorded without any buffer (pH 5). The guanine imino cross peaks are much weaker or completely missing in the magenta spectrum.

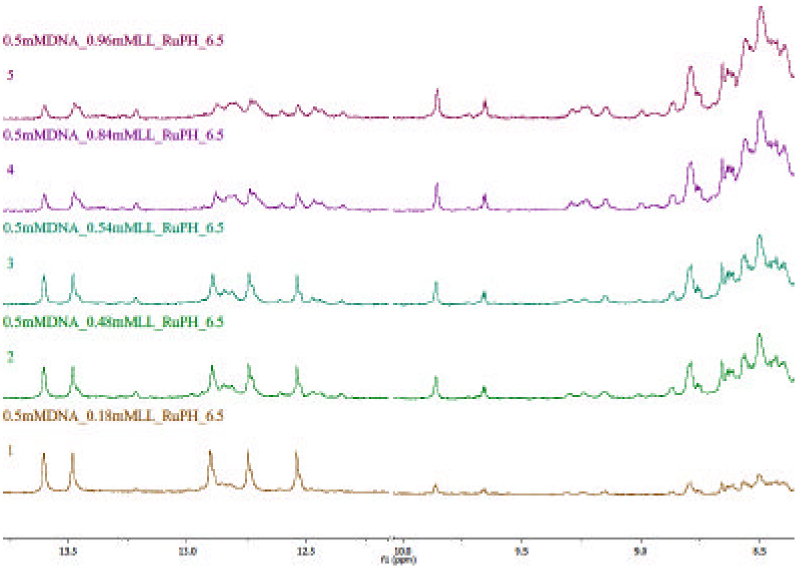

Figure S2. 1D-NMR titration of ? ,? -B into 0.5 mM DNA at 25^0, in 20mM pH 6.5 phosphate buffer solution, NaCl concentration: 100mM. When the ratio of [DNA]/[? ,? -B] exceeds 1:1, the line width of DNA imino protons become broadened. The ? ,? -B signals between 10-8 ppm remain unchanged by adding more ? ,? -B.

Section 2: Assignment of both symmetric and asymmetric DNA

Table S1 lists the chemical shifts for both free DNA and DNA binding ? ,? -B. For the latter, several protons on terminal base-pairs could not be assigned. All assignments were obtained in 20mM sodium phosphate buffer at 25^{0}C. Missing H3', H4', H5' and H5" protons overlap with the water signal. Figure S2 displays the chemical shift differences of strands a and ß compared to the free DNA. The ß-strand shows larger differences than the a-strand.

Table S1. Chemical shifts (ppm) of free d[(CGCGAATTCGCG)]$_2$ (on the lines with the nucleotide identification) and bound DNA (on the lines with "α" for the α-strand, "β" for the β-strand respectively).

Base	H8/H6	H2/H5	H1'	H2'	H2"	H3'	H4'	H3/H1	H41	H42
C_1	7.63	5.92	5.77	1.95	2.40	4.07	3.97			
a										
ß										
G_2	7.94	5.35	5.87	2.64	2.70	4.34				
a										
ß										
C_3	7.25		5.60	1.83	2.26	4.12	3.96		6.41	8.38
a	7.05		5.54	1.56	2.06					
ß	7.09			1.79	2.24					7.68
G_4	7.84		5.42	2.64	2.73		4.30	12.69		
a	7.72		5.36	2.61	2.79			12.58		
ß	7.77		5.52	2.53	2.63					
A_5	8.10	7.23	5.98	2.67	2.91	4.45	4.25			
a	8.04	7.13	5.93	2.64	2.83					
ß	8.62	7.14	6.02	2.64	2.90					
A_6	8.09	7.60	6.14	2.54	2.9	4.45	4.33			
a	8.05	7.40	5.84	2.42	2.73					
ß	8.56		6.33	2.44	2.92					
T_7	7.10		5.90	1.97	2.54	4.18		13.61		
a	6.94		5.67	1.83	2.42			13.48		
ß	6.74		5.63	2.02	2.35			13.40		
T_8	7.36		6.09	2.1	2.52	4.20	4.14	13.75		
a	7.26		5.97	2.05				13.57		
ß	7.21		6.06	1.91				13.38		
C_9	7.45	5.60	5.65	2.05	2.40	4.14	4.02		6.80	8.42
a	7.38		5.56	2.00	2.32					8.38
ß	7.43									
G_{10}	7.90		5.84	2.61	2.67	4.36		12.89		
a	7.83							13.34		
ß										
C_{11}	7.32	5.43	5.75	1.87	2.32	4.13			6.56	8.46
a										
G_{12}	7.94		6.15	2.34	2.60	4.14	4.06			

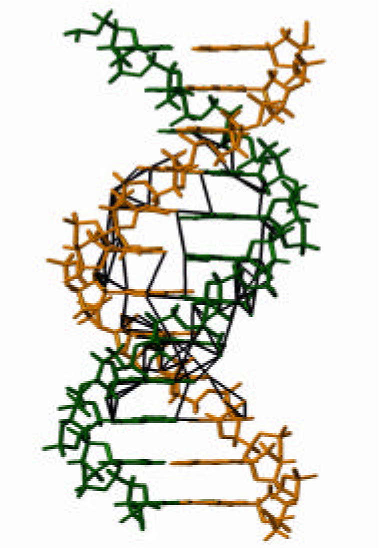

Figure S3. Network of observed NOEs indicated by black lines, of 1:1 DNA/Ru mixture. Green and orange color is used to discriminate strand a and strand ß respectively.

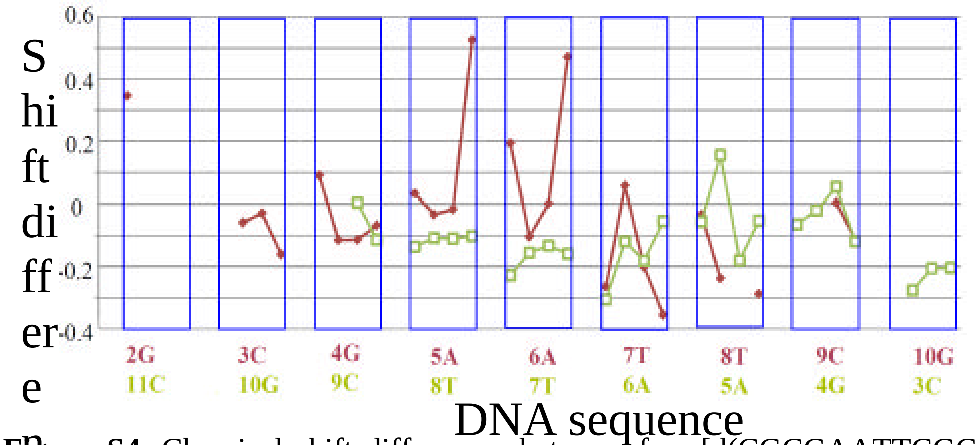

Figure S4. Chemical shift differences between free [d(CGCGAATTCGCG)]$_2$ and strand a (green), strand ß (red) as a function of the DNA sequence. Peaks inside each blue box are from left to right: H1', H2', H2" and H6/H8.

Section 3: Assignment of ? ,? -B and intermolecular NOEs

Assignment of resonances in free ? ,? -B are based on NOESY and TOCSY spectra (Table S2). For bound ? ,? -B, only four resonances can be identified; the remaining ones are covered by the DNA resonances. Two of these four show intermolecular NOEs: 9.14 ppm and 9.35 ppm (Fig. S3). The unambiguous assignment of these two resonances is explained below.

Table S2. Chemical shift of ? ,? -B in 20 mM phosphate buffer, pH 6.5.

**Proton**	**Chemical shift**
Dppz	
H6C/H6'C	8.38
H5C/H5'C	8.04
H4C/H4'C	9.86
H4D	8.30
H3D	7.94
H1D	8.43
BPY A	
H3	8.63
H4	8.12
H5	7.47
H6	7.89
H3'	8.57
H4'	8.00
H5'	7.21
H6'	7.55
BPY B	
H3	8.76
H4	8.25
H5	7.69
H6	8.28
H3'	8.61
H4'	8.15
H5'	7.48
H6'	7.96

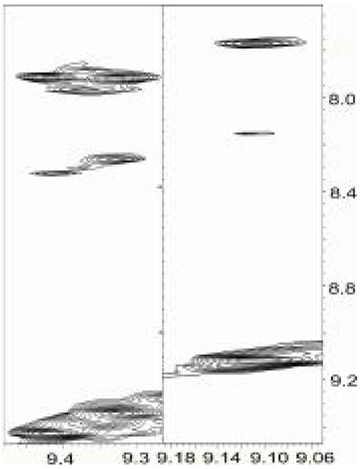

Figure S5. Extracts of the NOESY spectrum of a 1:1 mixture of ? ,? -B and [d(CGCGAATTCGCG)]$_2$. NOEs are shown for the following ? ,? -B resonances (horizontal axis): 9.35 ppm (left) and 9.14 ppm (right). All NOEs with shifts larger than 6.2 ppm (vertical axis) are shown, i.e. all intramolecular NOEs. All NOESY peaks (two on each side) coincide with TOCSY peaks.

Exclusion of assignment possibilities

Figure S3 shows specific patterns of cross peaks for the two resonances in question (9.14 and 9.35 ppm): two NOESY cross peaks that coincide with corresponding TOCSY peaks. From the nuclei with chemical shifts larger than 8.1ppm for the free ? ,? -B, H3D and H4D can be excluded because only one NOESY peak with a coinciding TOCSY peak is expected. Similarly, all H4, H6B, H6C and all H5 resonances should yield more than two NOESY/TOCSY peaks; overlap of peaks can be excluded due to the large shift difference for free ? ,? -B (>1 ppm), and/or due to NOESY peak intensities (very weak peaks for distances of 2.5Å).

This leaves the following protons as candidates for the four resonances >9 ppm: H4Ca, H4'Ca, H4Cb and H4'Cb. For all possible assignment combinations (six due to symmetry reasons), CYANA calculations were performed, yielding the results summarized in Table S3. The assignment H4'Cb to the resonance at 9.35 ppm and H4Ca to the resonance at 9.14 ppm results in negligible residual violations of constraints and a CYANA target function near zero.

Table S3. CYANA calculation for six possible assignments of the resonances at 9.35 and 9. 14 ppm

Assignment		CYANA target function	Maximal constraint violations (Å)	
9.14ppm	9.35ppm		Upper distance limits	Van der Waals limits
H4'Ca	H4Ca	30.84	3.67	0.73
H4Ca	H4'Ca	28.00	3.54	0.66
H4'Ca	H4'Cb	5.36	1.51	0.38
H4Ca	H4'Cb	0.2	0.14	0.13
H4'Ca	H4Cb	0.95	0.74	0.23
H4Ca	H4Cb	3.02	0.90	0.49

Section 4: Molecular dynamic Simulations

All Molecular Dynamics (hereafter MD) simulations were performed with the Amber11 software package [1]. FF-10 (in particular ff99bsc0 [2]) parameters were used for DNA, and GAFF parameters [3] for Λ-$[Ru(phen)_3]^{2+}$, excluding parameters of the ruthenium(II) ion coordination sphere (Ru-N bonds, Ru-N-C and N-Ru-N angles, and all torsion angles, which involve ruthenium). These latter parameters were calculated analogously to the procedure described in [4, 5] (compatible with GAFF force fields to be used with AMBER). RESP charges [6], geometry optimization of Λ,Λ-$[Ru(bipy)_4bidppz]^{4+}$ as well as Ru(II)-coordinating sphere parameters were obtained with the Gaussian09 software package [7] using B3LYP functional [8-10] with LANL2DZ basis set [11]. The prep file for the Λ,Λ-B ruthenium(II)-compound is available upon request.

MD simulations were performed according to the following protocol, using as a starting model a structure provided by CYANA calculations. The system (DNA and ruthenium(II)-complex, neutralized by sodium ions and solvated by 10 Å octahedron of explicit TIP3P waters [12]) was initially minimized by 1000 steps of steepest descent followed by 1000 steps of conjugate gradient, followed by fast heating (50 ps) from 0 to 300 K with the Langevin thermostat [13] temperature control scheme with collision frequency of 2 ps^{-1}, with harmonic restraints of 20 kcal/mol/$Å^2$ on the heavy atoms of the solutes, performed in constant volume. The restraints were then gradually reduced to zero in a series of equilibration runs of 100 ps each, at constant pressure (1 bar) and temperature (300 K) sustained using Langevin thermostat but with collision frequency of 1 ps^{-1}. The system was further equilibrated using steered MD (based on intermolecular NOE restraints) during 2 ns, followed by productive, unrestrained MD trajectories of 5 ns, both recorded with the following parameters. An integration time step of 2 fs was used and all bond lengths involving hydrogen atoms were constrained using SHAKE [14]. Long-range interactions were treated using the PME approach with a 10 Å direct space cut-off. Steered MD run was coupled to Replica Exchange MD for better sampling of the conformational space. Taking into account the properties of DNA as well as that all simulations were performed with explicit solvent model, the temperature span was only 9 degrees (F), starting from 300 to 309 K, resulting in total 10 replicas for each of the steered MD runs. The fully unrestrained MD trajectories of 5 ns length recorded for the ten replica were used to represent the structure of the DNA-? ,? -B complex.

References

[1] Case, D.A., Darden, T. A., Cheacham, T. E., Simmerling, C. L., Wang, J., Deke, R.E.,Luo,R., Walker, R.C.,Zhang, W., and Merz, K.M. (2010) AMBER 11, University of California, San Francisco.

[2] Pérez, A., Marchán, I.,Svozil, D.,Sponer, J., Cheatham III, T.E., Laughton, C.A. and Orozco, M. (2007) Refinement of the AMBER force field for nucleic acids: improving the description of alpha/gamma conformers. *Biophys. J.*, **92**, 3817-3829.

[3] Wang, J. M., KollmanP.A, W., and Case, D. A. (2004) Development and testing of a general amber force field. J. Comput. Chem., **25**, 1157-1174.

[4] Norrby, P.-O., and Liljefors T. (1997) Automated Molecular mechanics parameterization with simultaneous utilization of experimental and quantum mechanical data. J. Comput. Chem., **19**, 1146-1166.

[5] Brandt,P., Norrby, T., Åkermark, B. and Norrby, P.-O. (1998) Molecular mechanics (MM3*) parameters for ruthenium (II)-polypyridyl complexes. Inorg. Chem., **37**,4120-4127.

[6] Bayly, C. I., Cieplak, P., Cornell, W. and Kollman, P. (1993) A well-behaved electrostatic potential based method using charge restraints for deriving atomic charges: the RESP model, *J. Phys. Chem.*, **97**, 10269-10280.

[7] Frisch, M. J., et al., (2003) Gaussian 03 (Gaussian, Inc., Wallingford, CT, 2003).
[8] Lee, C., Yang, W. and Parr, R. G. (1988) Development of the Colle-Salvetti correlation-energy formula into a functional of the electron density. *Physic. Rev. B*, **37**, 785-789.
[9] Becke, A. D. (1993) A new mixing of Hartree-Fock and local density-functional theories. *J. Chem. Phys.*, **98**, 1372.
[10] Stephens, P. J., Devlin, F. J., Chabalowski, C. F. and Frisch, M. J. (1994) Ab Initio Calculation of Vibrational Absorption and Circular Dichroism Spectra Using Density Functional Force Fields. *J. Phys. Chem.*, **98**, 11623-11627.
[11] Hay, P. J. and Wadt, W. R. (1985) Ab initio effective core potentials for molecular calculations. Potentials for the transition metal atoms Sc to Hg. *J. Chem. Phys.*, **82**, 270.
[12] Jorgensen, W.L., Chandrasekhar, J., Madura, J.D., and Klein, M.L. (1983) Comparison of simplex potential functions for simularting liquid water. J. Chem. Phys., **79**, 926-935.
[13] Izaguirre, J. A., Catarello, D. P., Wozniak, J. M.. and Skeel, R. D. (2001) Langevin stabilization of molecular dynamics. *J. Chem. Phys.*, **114**, 2090.
[14] Ryckaert, J.-P., Ciccotti, G. and Berendsen, H.J.C. (1977) Numerical integration of the Cartesian equations of motion of a system with constraints: Molecular dynamics of n-alkanes. *J. Comput. Phys.*, **23**, 327–341.

Section 5: Other interaction modes

In contrast to natural long DNA, our system consists of a short DNA fragment. The present experimental observations suggest that the terminal DNA base pairs of the symmetric DNA (i. e. of a binding mode different from the minor groove binding state of ? ,? -B) interact with the ? ,? -B in fast exchange process (Fig. S5). This may be explained by a hydrophobic interaction due to the stacking of the aromatic ring system of the ? ,? -B with the terminal DNA bases.

When varying the temperature, it was also discovered that some resonances of ? ,? -B were not temperature dependent, while others were reduced with increasing temperature, together with those of the melting DNA (melting temperature *ca*. 50°C; Fig. S6). Comparison with a non-buffered sample showed that the temperature-independent peaks are correlated to the presence of phosphate buffer, suggesting a buffer-mediated aggregation of ? ,? -B and oligomer, which observation was not investigated further (buffered and non-buffered NOESY spectra are compared for ? ,? -B shown in Fig. S6a).

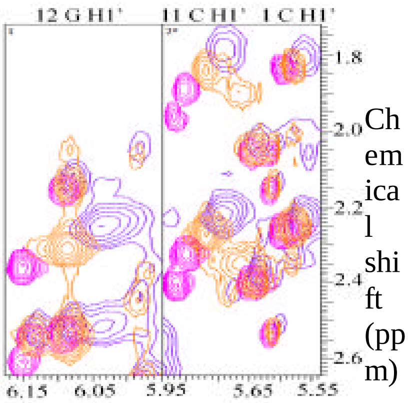

Chemical shift (ppm)

Chemical shift (ppm)

Figure S6. Fast exchange evidence for ? ,? -B and DNA: Overlay of three ^{1}H NOESY spectra (t_m =100 ms): 1mM DNA-only (magenta), 0.5mM ? ,? -B mixed with 1mM DNA (orange); mixture of 1mM ? ,? -B and 1mM DNA (blue).

Comparison between NOESY spectra of DNA-? ,? -B with and without buffer. The presence of buffer results in additional peaks that are temperature independent; some of these are shown in the red boxes in Fig. S6. A few more are found between 7 and 9 ppm; these peaks make no NOE contacts except to the resonances shown in the red boxes of Fig. S6.

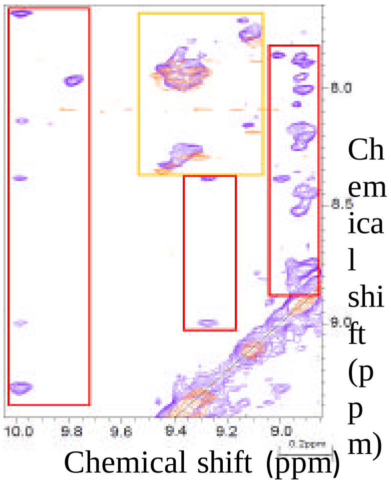

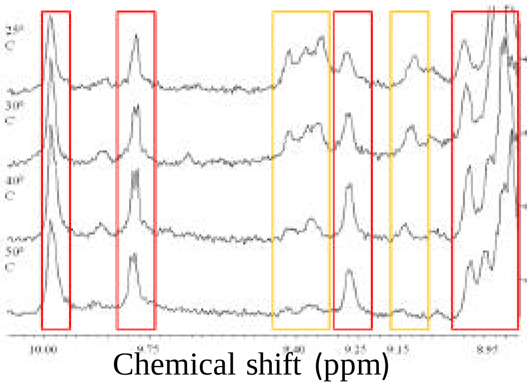

FigureS7a: Overlay of NOESYs with (purple) and without (orange) buffer. A region with only ? ,? -B signals is shown. Both spectra were recorded at 25 ^{0}C.

Figure S7b. Temperature independent (red boxes) and temperature dependent peaks (yellow boxes).
